# Modulation of the stress-activated protein kinases Hog1/p38 and a TORC1-dependent kinase by curcumin is stress granule-dependent

**DOI:** 10.1016/j.jbc.2026.111402

**Published:** 2026-03-25

**Authors:** Liuyi Zheng, Zenae K. Cherry, Gabriela Soltys, Marisa Penner, Natalie Samuell, Dini Welivita, Brianna Thomas, Jonathan S. Fisher, Yuqi Wang

**Affiliations:** Department of Biology, Saint Louis University, St Louis, Missouri, USA

**Keywords:** curcumin, stress granule, Hog1, p38, S6, yeast, L6 cells

## Abstract

Curcumin, a natural polyphenolic compound, is widely recognized for its anti-inflammatory, anticancer, and antimicrobial properties. Despite its long history of medicinal use, the precise mechanisms underlying its cellular effects remain incompletely understood. In yeast, curcumin has been shown to activate Hog1, a stress-activated protein kinase (SAPK), but the regulatory basis of this activation is unclear. Here, we investigated the role of stress granules in mediating the effects of curcumin. We found that either disrupting Pub1, a core stress granule protein in yeast, or applying the stress granule dissolution agent lipoamide markedly reduced curcumin-induced Hog1 activation. Curcumin is also known to inhibit S6 phosphorylation mediated by a TORC1-dependent kinase in mammals. We found that this phenomenon occurs in yeast as well, and the effect was similarly dependent on Pub1 and lipoamide. Using the rat skeletal muscle cell line L6, we showed that lipoamide likewise dampens the effect of curcumin on p38 activation. Together, these findings identify stress granules as a critical mediator of the effects of curcumin on SAPK and S6 signaling, providing new insight into how this ancient compound modulates cellular signaling pathways.

Curcumin is a natural polyphenolic compound derived from the rhizome of the plant *Curcuma longa* (1, 2, 3). Traditionally used as a spice and coloring agent in culinary practices, it has emerged as a fascinating compound with various pharmacological properties, including anti-inflammatory (1, 4, 5), antioxidant (6, 7), anticancer (8, 9, 10), and antimicrobial effects (11, 12, 13). Studying the mechanisms of action of curcumin is important due to its broad spectrum of bioactivities and its potential in developing novel therapeutic strategies. While curcumin has been studied in mammalian systems, investigating its actions in simpler model organisms provides valuable insights into conserved biological processes and molecular mechanisms. Budding yeast *Saccharomyces cerevisiae* is a well-established model organism for understanding basic biological processes and has been instrumental in uncovering fundamental principles of eukaryotic biology (14, 15, 16). The genetic tractability, well-defined signaling pathways, and conservation of key cellular processes between yeast and higher eukaryotes make it an excellent model to study the effects of curcumin and elucidate its molecular targets (17).

It is known that curcumin treatment in yeast leads to an activation of Hog1 (18), a stress-activated or mitogen-activated protein kinase with p38 as its mammalian homolog (19). However, thae underlying mechanism by which curcumin activates Hog1 remains largely unknown. A very elegant recent work has demonstrated that Hog1 activation induced by acetic acid is dependent on stress granules (20), which are dynamic, non-membranous cytoplasmic aggregates of proteins and RNAs that form in response to various cellular stressors such as dsRNA, ER overload, amino acid starvation, arsenate, and redox stress (21, 22). Stress granules have emerged as critical regulators of cell signaling pathways, influencing processes such as apoptosis, immune responses, and cell survival (23). The formation and dissolution of stress granules are tightly regulated, and dysregulation has been implicated in various diseases, including neurodegeneration and cancer (21, 24). In this study we have examined if stress granules are involved in mediating the effect of curcumin in activating Hog1 in yeast, using both genetic and pharmacological interventions. We find that disrupting stress granule dynamics severely impairs the ability of curcumin to activate Hog1. We also find that curcumin inhibits phosphorylation of S6, an event downstream of TORC1 signaling in yeast (25), and the effect is likewise dependent on stress granule dynamics. Notably, lipoamide abolishes the effect of curcumin on p38 in L6 cells, which are a cell line derived from rat skeletal muscle (26) and known to induce p38 activation by curcumin (27). We suggest that stress granules are critical for the effects of curcumin on SAPK and TORC1-dependent phosphorylation of S6.

## Results

### Curcumin-induced activation of Hog1 is dependent on stress granules

**Figure 1:**
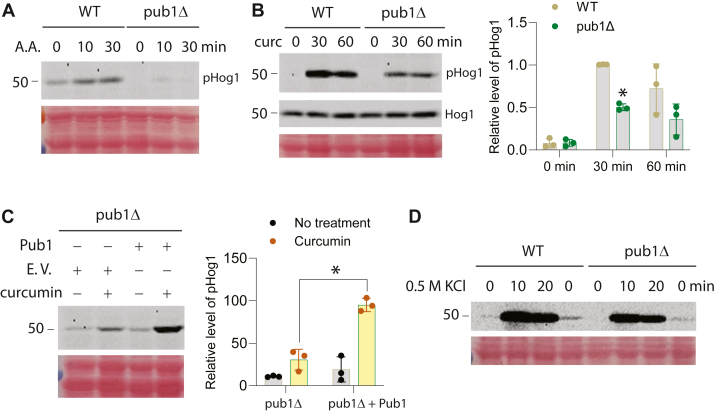
**Hog1 activation by curcumin is dependent on Pub1 and modulated by lipoamide.***A*, wild-type (WT) or the *pub1Δ* cells were grown in YDP (pH 4.5) to mid-log phase and then shifted to YPD with 100 mM acetic acid (pH 4.5) for the indicated time (20). Whole cell extracts were separated on 8% SDS-PAGE and immunoblotted with anti-phospho-p38 to detect phosphorylated and activated Hog1 (p-Hog1). Equal loading was confirmed with ponceau staining. *B*, WT or the *pub1Δ* cells were grown in YDP to mid-log phase and then treated with curcumin for the indicated time. Whole cell extracts were separated on 8% SDS-PAGE and immunoblotted with anti-phospho-p38 to detect phosphorylated and activated Hog1 (p-Hog1) and with anti-Hog1 to detect total Hog1. Equal loading was confirmed with ponceau staining. The quantified data from three independent experiments were shown on the *right. C*, the *pub1Δ* cells transformed with either pRS315-*PUB1* (Pub1 in the figure) or an empty vector (E.V. in the figure) were grown to mid-log phase, treated or not treated with curcumin for 30 min. Whole cell extracts were separated on 8% SDS-PAGE and immunoblotted with anti-phospho-p38 to detect phosphorylated and activated Hog1 (p-Hog1). Equal loading was confirmed with ponceau staining. The quantified data from three independent experiments were shown on the *right. D*, WT or the *pub1Δ* cells were grown in YDP to mid-log phase and then treated with 0.5 M KCl for the indicated time. Whole cell extracts were separated on 8% SDS-PAGE and immunoblotted with anti-phospho-p38 to detect phosphorylated and activated Hog1 (p-Hog1). Equal loading was confirmed with ponceau staining.

Curcumin treatment is known to activate Hog1 (18), but the underlying mechanism has not been established. As Hog1 activation can be dependent on stress granules in response to acetic acid exposure (20), we asked if curcumin-induced Hog1 activation is similarly dependent on stress granules. To this end, we made use of the *pub1Δ* mutant, which lacks the poly(U)-binding protein Pub1 and is known to disrupt the dynamics of stress granules (28). We first compared the acetic acid-induced Hog1 activation in wild type and the *pub1Δ* mutant to verify the effect of the *pub1Δ* mutant. Hog1 activation was measured by monitoring the level of phosphorylated and thus activated Hog1. As shown in Figure 1*A*, acetic acid induces a mild activation of Hog1, and the effect is markedly reduced in the *pub1Δ* mutant, consistent with the report that stress granules are required for acetic acid-induced Hog1 activation (20). We then examined the effect of curcumin. As shown in Figure 1*B*, curcumin induces a substantial activation of Hog1, and its extent of activation is drastically reduced in the *pub1Δ* mutant. This effect is not due to an alteration of Hog1 abundance, as the same level of total Hog1 protein is present in both wild-type cells and the *pub1Δ* mutant (Fig. 1*B* middle panel). Importantly, the activation of Hog1 with curcumin treatment is restored after a plasmid-borne Pub1 is introduced into the mutant. This indicates that decreased Hog1 activation in the *pub1Δ* mutant is indeed due to the loss of Pub1 (Fig. 1*C*). Notably, hyperosmotic stress-induced Hog1 activation still robustly occurs in the *pub1Δ* mutant (Fig. 1*D*).

**Figure 2:**
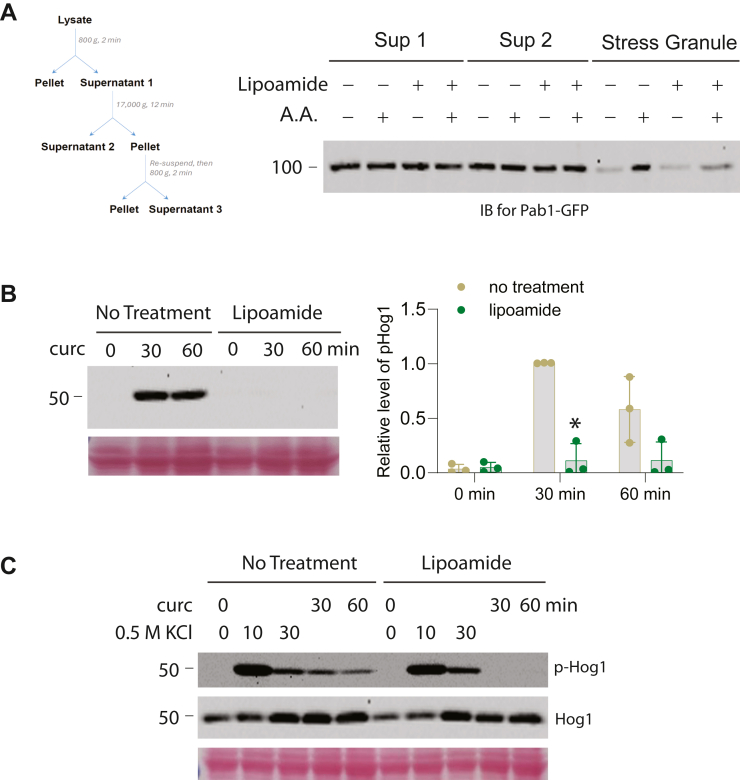
**Hog1 activation by curcumin is dependent on Pub1 and modulated by lipoamide.***A*, cells expressing Pab1-GFP were grown to mid-log phase, pre-treated or not with 100 μM lipoamide for 1 h and then treated with acetic acid for 30 min. Whole cell lysates were subjected to differential centrifugation and examined for the presence of Pab1-GFP. The supernatant three fraction in the flow chart corresponds to stress granule. *B*, mid-log phase wild type cells were pre-treated with 100 μM lipoamide or not for 1 h and then treated with curcumin for the indicated time. Whole cell extracts were separated on 8% SDS-PAGE and immunoblotted with anti-phospho-p38 to detect phosphorylated and activated Hog1 (p-Hog1). Equal loading was confirmed with ponceau staining. The quantified data from three independent experiments are shown on the *right*. *C*, mid-log phase wild type cells were pre-treated with 100 μM lipoamide or not for 1 h and then treated with either curcumin or KCl for the indicated time. Whole cell extracts were separated on 8% SDS-PAGE and immunoblotted with anti-phospho-p38 to detect phosphorylated and activated Hog1 (p-Hog1) or anti-Hog1 to detect the total Hog1. Equal loading was confirmed with ponceau staining.

As an alternative strategy to disrupt the dynamics of stress granules, we chose to treat cells with lipoamide, a chemical recently identified as an agent that is capable of dissolving stress granules, thus impairing their dynamics (29). To confirm that lipoamide does affect stress granule formation in yeast, we made use of the well-established stress granule marker Pab1-GFP to monitor the formation of stress granules (20, 30). We isolated stress granules from cells expressing Pab1-GFP using differential centrifugation, following an established procedure (Fig. 2*A*) (20, 30) and examined the presence of Pab1-GFP in the stress granule fraction. As shown in Figure 2*A*, acetic acid treatment induces an accumulation of Pab1-GFP in the stress granule fraction, and the effect is clearly diminished by the pre-treatment of cells with lipoamide, indicating that lipoamide does impair stress granule formation as expected. We then examined whether lipoamide treatment has any effect on curcumin-induced Hog1 activation. As shown in Figure 2*B*, lipoamide treatment severely blocked the effect of curcumin in activating Hog1. This effect appears to be specific, as lipoamide treatment does not prevent hyperosmolarity-induced Hog1 activation (Fig. 2*C*). It is worth noting that the abundance of Hog1 protein is not clearly affected by lipoamide treatment. Collectively, both genetic disruption (*pub1Δ*) and pharmacological inhibition (lipoamide) of stress granule dynamics impair curcumin-induced Hog1 activation, suggesting that Hog1 activation by curcumin is stress granule-dependent.

### Curcumin affects yeast S6 kinase signaling in a stress granule-dependent manner

**Figure 3:**
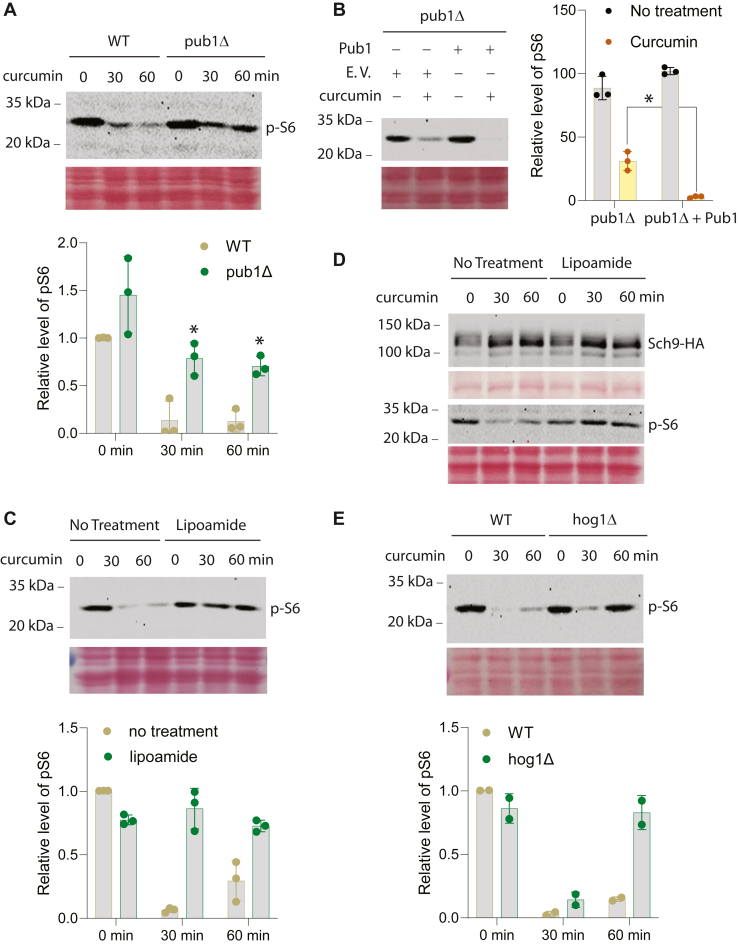
**Inhibition of S6 phosphorylation by curcumin is partially dependent on Pub1 and modulated by lipoamide.***A*, WT and the *pub1Δ* cells were grown to mid-log phase and treated with curcumin for the indicated time. Western blot analysis was conducted for phosphorylation of S6 ribosomal protein (p-S6). Equal loading was confirmed with ponceau staining. The quantified data from three independent experiments are shown at the bottom. *B*, the *pub1Δ* cells were transformed with either pRS315-*PUB1* (Pub1 in the figure) or an empty vector (E.V. in the figure). Mid-log phase cells were treated or not treated with curcumin for 30 min, and whole cell extracts were analyzed by Western blot for phosphorylation of S6 ribosomal protein (p-S6). Equal loading was confirmed with ponceau staining. The quantified data from three independent experiments are shown on the *right*. *C*, WT cells were grown to mid-log phase, pre-treated or not with 100 μM lipoamide for 1 h, and then treated with curcumin for the indicated time. Whole-cell extracts were analyzed by Western blot for phosphorylation of S6 ribosomal protein (p-S6). Equal loading was confirmed with ponceau staining. The quantified data from three independent experiments are shown at the *bottom*. *D*, WT cells transformed with a plasmid that expresses Sch9-HA were grown to mid-log phase, pre-treated or not with 100 μM lipoamide for 1 h and then treated with curcumin for the indicated time. Whole cell extracts were analyzed by Western blot for Sch9-HA and phosphorylation of S6 ribosomal protein (p-S6). Equal loading was confirmed with ponceau staining. *E*, WT and the *hog1Δ* cells were grown to mid-log phase and treated with curcumin for the indicated time. Western blot analysis was conducted for phosphorylation of S6 ribosomal protein (p-S6). Equal loading was confirmed with ponceau staining. The quantified data from repeated experiments were shown at the *bottom*.

We then set out to examine if the stress granule dependency of curcumin effects is specific to Hog1. To this end, we chose to investigate target of rapamycin (TOR) signaling, which is critical for cell growth and diseases, including various cancers (31). Curcumin is known to inhibit the TORC1 branch of TOR signaling in mammalian cells (32). Consistent with this finding, curcumin treatment in yeast substantially reduces the level of phosphorylated ribosomal protein S6 (pS6, Fig. 3*A*), which has been documented as a reliable marker for TORC1 signaling in yeast (25). Interestingly, the effect of curcumin on pS6 is clearly diminished in the *pub1Δ* mutant (Fig. 3*A*), and putting Pub1 back into the *pub1Δ* mutant restores the more pronounced inhibition of pS6 (Fig. 3*B*). Likewise, lipoamide treatment substantially blocked the effect of curcumin in reducing the level of S6 phosphorylation (Fig. 3*C*). In addition to S6 phosphorylation, which is primarily catalyzed by the AGC kinase Ypk3 in yeast (25), phosphorylation of Sch9, a major target of TORC1 in yeast (33), is often used as another readout of TORC1 activity. Accordingly, we used an HA-tagged version of Sch9 and examined its phosphorylation. As shown in Figure 3*D*, Sch9-HA migrates as multiple species due to differential phosphorylation, with slower-migrating bands corresponding to more heavily phosphorylated forms (33). Upon curcumin treatment, a mobility shift toward faster-migrating (hypo-phosphorylated) species was observed, which is consistent with an inhibition of TORC1 by curcumin. However, lipoamide treatment does not impair the ability of curcumin to reduce Sch9-HA phosphorylation. Re-probing the same samples with anti-pS6 again showed that the effect of curcumin on pS6 is abolished by lipoamide. These findings indicate that stress granules are required for the full inhibitory effect of curcumin on one branch of TORC1 signaling, *i.e.*, Ypk3 activation, but not the other branch of TORC1 signaling, *i.e.*, Sch9 activation, in yeast.

It is possible that the effects of curcumin on Hog1 activation and Ypk3 inhibition are linked, in a manner like a previous report that hyperosmolarity-induced TORC1 signaling inhibition requires Hog1 (34). We thus examined whether Hog1 is required for the effect of curcumin on S6 phosphorylation. For this, we examined the effect of curcumin on S6 phosphorylation in the *hog1Δ* mutant. As shown in Figure 3*E*, deletion of *HOG1* gene initially modestly reduced the effect of curcumin on pS6, but it nearly abolished the effect of prolonged curcumin effect on pS6, indicating that Hog1 represses late-stage S6 phosphorylation in response to curcumin.

### Curcumin treatment on stress granule formation

**Figure 4:**
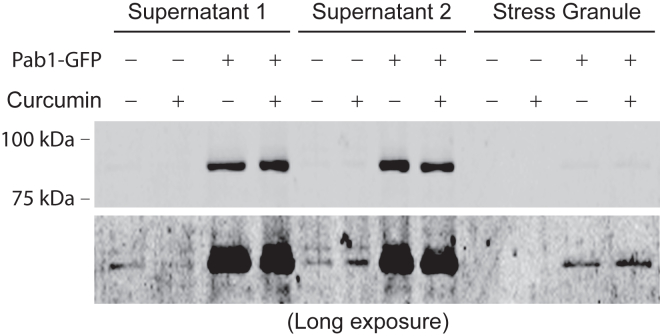
**The effects of curcumin on stress granules.** Cells expressing Pab1-GFP were grown to mid-log phase, treated with either DMSO or curcumin for 1 h. Whole cell lysates were subjected to differential centrifugation and examined for the presence of Pab1-GFP. The supernatant three fraction corresponds to the stress granule.

Having demonstrated that the effect of curcumin on both Hog1 and one branch of TOR signaling is dependent on stress granules, we next sought to examine whether curcumin treatment induces stress granule formation. To this end, we isolated stress granules from cells expressing Pab1-GFP using differential centrifugation, following an established procedure (Fig. 2*A*) (20, 30), and examined the presence of Pab1-GFP in the stress granule fraction. Only very faint signals, which presumably represent a basal level of stress granules, were detected, and curcumin treatment did not make a clear difference (Fig. 4). This result suggests that curcumin treatment does not influence the level of stress granules *per se*, and it is possible that it can influence the dynamics of stress granule formation, which was not detected due to the technical limitations of the method.

### Curcumin effects in L6 cells

**Figure 5:**
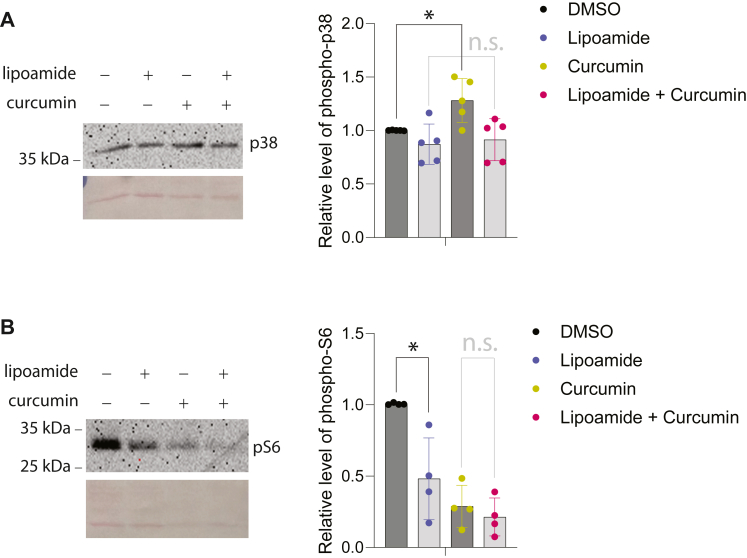
**L6 myotube cells were treated with either lipoamide, or curcumin, or both as indicated.** Whole-cell lysates were subjected to Western blot analysis for phosphor-p38 (*panelA*) or phosphor-S6 (*panelB*). Equal loading was confirmed with ponceau staining. The quantified data from multiple independent experiments were shown on the *right* of each panel. ∗ denotes the statistical significance with a *p* value less than 0.05.

To determine if our finding that lipoamide impairs the effects of curcumin on Hog1 activation and S6 phosphorylation is applicable in mammalian cells, we decided to test the effects of lipoamide and curcumin in L6 cells, which were derived from rat skeletal muscle (26). L6 cells were chosen because a previous study showed that curcumin induces p38 activation in this cell line (27), similar to what we have observed in yeast. As shown in Figure 5*A*, curcumin indeed induces a mild activation of p38 with a similar modest magnitude as reported previously (27). Interestingly, the effect of curcumin in activating p38 is largely abolished by pretreatment with lipoamide. We also examined the level of S6 phosphorylation in L6 cells. As expected from the inhibitory effect of curcumin on TOR signaling in mammalian cells (32), curcumin treatment drastically reduces the level of S6 phosphorylation (Fig. 5*B*). Notably, lipoamide treatment itself also significantly reduces the level of S6 phosphorylation, while it does not further reduce the level of S6 phosphorylation in curcumin-treated cells. Thus, in the presence of lipoamide, curcumin has less of an effect on S6 phosphorylation.

## Discussion

Curcumin is a pleiotropic natural product with many medicinally related antimicrobial, anti-inflammatory, antioxidant, and anticancer properties (7, 12, 35, 36). Despite its broad activity, we still do not fully understand the cellular mediators that couple curcumin exposure to changes in stress-activated protein kinase and target of rapamycin signaling. In this work, using budding yeast and rat L6 myotubes, we reveal that stress granule integrity is a key factor of the effects of curcumin on the SAPK Hog1/p38 and on the Ypk3/S6 branch of TORC1 signaling. In yeast, genetic disruption of stress granule dynamics by deleting the *PUB1* gene or pharmacological dissolution of stress granules with lipoamide, markedly impairs the ability of curcumin in activating Hog1 and in inhibiting S6 phosphorylation; in mammalian L6 cells, lipoamide also attenuated the impact of curcumin on p38 activation. Notably, the magnitude of the curcumin-induced signaling effects in L6 cells is more modest than in yeast. These findings identify stress granules as a previously underappreciated mediator linking curcumin to conserved stress-response pathways.

**Figure 6:**
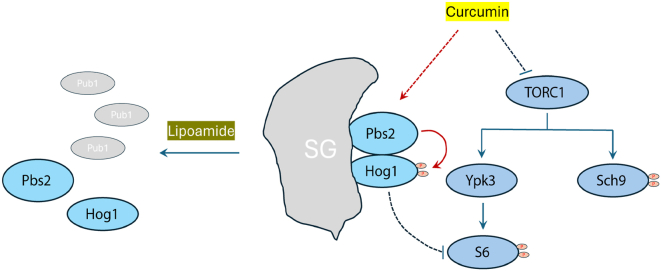
**A model that explains the findings. Stress granules (SGs) concentrate or position the core MAPK module, *e.g.*, MAPKK Pbs2 and MAPK Hog1, to facilitate signal transmission under curcumin treatment.** Lipoamide dissolves stress granules and thus impairs curcumin-induced Hog1 activation. Hog1 is partially responsible for the effect of curcumin-induced inhibition of S6 phosphorylation, and thus lipoamide could block curcumin-induced S6 phosphorylation by preventing Hog1 activation. Sch9 phosphorylation downstream of TORC1 is independent of SGs.

Our study extends recent findings that Hog1 activation by acetic acid in yeast requires stress granule function (20) by demonstrating that curcumin-induced Hog1 activation is similarly stress granule-dependent. A working model is that stress granules concentrate or position the core MAPK module, *e.g.*, MAPKK Pbs2 and MAPK Hog1, to facilitate signal transmission under certain stress conditions (Fig. 6). In such a scenario, stress granules would act not merely as storage depots but as dynamic platforms that enhance proximity, local stoichiometry, or post-translational modification states of signaling components. The observation that lipoamide, which dissolves stress granules (29), strongly reduces Hog1 activation supports the idea that stress granule integrity is needed for SAPK activation by curcumin. The extension to mammalian p38 activation in L6 cells indicates that stress granule-linked SAPK control is conserved across eukaryotes.

One interesting finding of our study is the branch-specific requirement for stress granules within TOR signaling. Curcumin robustly decreased phosphorylation of S6 in yeast, a modification largely driven by the AGC kinase Ypk3 (25), and the effect was dependent on stress granules. In contrast, the impact of curcumin on the TORC1 target Sch9 as assessed by its mobility shift persisted even when stress granules were dissolved. These observations suggest that Ypk3/S6 signaling is spatially and functionally closer to stress granule-regulated machinery than Sch9, which may rely on distinct localization, *e.g.*, the vacuolar membrane (37, 38, 39), and regulatory inputs. Stress granules could influence Ypk3/S6 signaling either by partitioning ribosomal components and translation factors that feed into S6 phosphorylation, or by recruiting modulators such as kinases, phosphatases, or scaffolds that preferentially control the Ypk3 branch. The Sch9 axis, by contrast, appears to be independent of stress granules, consistent with differential compartmentalization of TORC1 outputs (39) and providing a mechanistic rationale for the selective sensitivity of downstream branches to stress granule integrity.

Deletion of *HOG1* partially reduced the early effect of curcumin on S6 phosphorylation and almost abolished the prolonged effect, implicating Hog1 in sustaining S6 inhibition. The temporal distinction, *i.e.*, modest early impact but strong late dependence, suggests a potentially dual mechanism. One mechanism is a stress granule-proximal, Hog1-independent component that rapidly depresses S6 phosphorylation, and another is Hog1-dependent component that consolidates or amplifies the effect over time. The mammalian data, where lipoamide reduces p38 activation and lowers S6 phosphorylation, supports the idea that p38 activity can contribute to sustained repression of S6 in response to curcumin.

Despite the functional requirement for stress granules, our microscopy and biochemical fractionation did not reveal pronounced changes in stress granule abundance following curcumin treatment. This apparent discrepancy can be rationalized if curcumin primarily affects the dynamics, composition, or nanoscale organization of stress granules, rather than bulk number or size. Subtle shifts such as altered dwell times of signaling proteins within stress granules, changes in stress granule subdomains, or selective recruitment and/or expulsion of regulators, could profoundly impact signaling yet remain below the detection threshold of our conventional light microscopy due to the diffraction limit (30, 40). Live-cell imaging and super-resolution approaches could possibly resolve such dynamic features. Another possibility is that curcumin could impact SAPK activation *via* post-transcriptional mechanisms in a stress granule-dependent manner. Stress granules are known to serve as a site for storing messenger RNAs and regulate their translation and decay (41). Nuclear Hog1 localization is sometimes used as a proxy for activated Hog1 (42). Studies using an elegant combination of microfluidics and time-lapse microscopy have demonstrated that stress-induced nuclear Hog1 localization can be influenced by P-bodies and stress granules through regulation of mRNA fate for stress-response genes (43). It would be interesting to investigate in the future if curcumin affects the localization of these genes in stress granules and/or P-bodies, which in turn leads to an activation of Hog1.

Taken together, our findings suggest that the effects of curcumin on Hog1/p38 activation and Ypk3/S6 inhibition require intact stress granule dynamics, whereas Sch9 regulation by curcumin proceeds largely independent of stress granules. Our study underscores stress granules as critical mechanistic mediators of the signaling actions of curcumin and condensate-centered strategies for fine tuning stress and growth pathways in yeast and in mammalian cells.

## Experimental procedures

### Strains and plasmids

Standard methods for the growth, maintenance, and transformation of yeast and bacteria and for the manipulation of DNA were used throughout. The yeast *S. cerevisiae* strains used in this study are BY4741 (*MATa leu2Δ met15Δ his3Δ ura3Δ*) and BY4741-derived mutant lacking *PUB1* (Research Genetics). The pRP1657 plasmid that expresses Pab1-GFP and Edc3-mCherry was a generous gift from Dr Roy Parker (44). A plasmid that expresses Pub1 was constructed by PCR amplifying the *PUB1* gene and subcloning it to the XmaI and XbaI sites of the pRS315 vector. The primers are 5′-TCC CCC GGG GGT GGT TCT GGC ACA CAT TT-3′ and 5′-GCT CTA GAG CTG TTG CCT TTA TGT GCC T-3′. A plasmid that expresses HA-tagged Sch9 was constructed by PCR amplification of the *SCH9* gene and subcloning it to the HindIII and XbaI sites of the pRS315 vector. The primers are 5′-ATG AAC GAT TTC GTT ACC CTC GG-3′ and 5′-CTA GTC TAG ATA TTT CGA ATC TTC CAC TGA CAA AT-3′. The HA tag was amplified using the following primers 5′-CTA GTC TAG ATA CCC ATA CGA TGT TCC TGA CTA TG-3′ and 5′-ATA AGA ATG CGG CCG CAG CAC TGA GCA GCG TAA TCT G-3′, and subcloned into the XbaI and NotI sites of the pRS315-*SCH9* generated above.

### L6 myotube cells

An L6 rat myoblast cell line was obtained from American Type Cell Culture Collection (ATCC). Cell culture procedures were followed as described by Somwar *et al.* with minor modifications (45). The L6 myoblasts were cultured in low-glucose Dulbecco’s Modified Eagle’s Medium (DMEM, Millipore-Sigma) supplemented with 10% fetal bovine serum (Gemini Bioscience) and 1% penicillin-streptomycin (Millipore-Sigma) at 37 °C in 5% CO_2_. Cells were passaged every 2 to 3 days until 70% confluency was obtained. L6 myoblasts were then seeded into 12-well culture plates 2 days prior to differentiation into myotubes in differentiation medium (DMEM, 2% horse serum from Millipore-Sigma and 1% penicillin-streptomycin) at 37 °C at 5% CO_2_. The cells received experimental treatments 48 h after differentiation, when myotube formation was observed. L6 myotubes were first treated with 80 μM lipoamide dissolved in DMSO or a DMSO vehicle for 60 min. The myotubes were then exposed to 100 μM curcumin dissolved in DMSO or a DMSO vehicle for 30 min. Following the treatments, the myotubes were washed twice with HEPES-buffered saline (HBS) (20 mM HEPES-Na (pH 7.4), 140 mM NaCl, 5 mM KCl, 2.5 mM MgSO_4_, and 1 mM CaCl_2_), before being scraped in lysis buffer (50 mM HEPES (pH 7.4), 150 mM NaCl, 10% glycerol, 1% Triton X-100, 1.5 mM MgCl_2_, 1 mM EDTA, 10 mM Na_4_P_2_O_7_, 100 mM NaF, 2 mM Na_3_VO_4_, 10 μg/ml aprotinin, 10 μg/ml leupeptin, 0.5 ug/ml pepstatin, and 0.2 mM phenylmethlysulfonyl fluoride (PMSF)). The lysates were then collected and centrifuged at 4 °C at 13,500 rpm for 10 min. The supernatant from the lysed and centrifuged cells was then collected, and a bicinchoninic acid (BCA) assay (Thermo Fisher) was performed to quantify protein concentrations, and samples were subjected to Western blot analysis for the presence of phosphor-p38 and pS6.

### Stress granule isolation

Stress granule isolation was conducted as described previously (20, 30). Briefly, wild-type cells were transformed with the pRP1657 plasmid that expresses Pab1-GFP. Mid-log phase cells with a volume of 50 ml were treated or not treated with 100 μM curcumin for 1 h, and cell pellets were resuspended in lysis buffer (50 mM Tris-HCl, pH 7.5, 150 mM NaCl, 5 mM EDTA, 25 mM NaF, 25 mM glycerophosphate, 1 mM PMSF, and EDTA-free protease cocktail). After rigorous vortexing in the presence of glass beads for 30 s for 6 times, the lysates were centrifuged at 800*g* for 2 min. The supernatants were further centrifuged at 17,000*g* for 12 min, and the pellets containing stress granules were washed with lysis buffer. After the final centrifugation at 800*g* for 2 min, the supernatants were collected as the isolated stress granule and subjected to Western blot analysis for the presence of Pab1-GFP.

### Western blotting

For the immunoblotting analysis, mid-log phase cells were grown on appropriate medium and then treated or not treated with 100 μM curcumin for 1 h as indicated. Curcumin was dissolved in DMSO. Proteins were extracted *via* trichloroacetic precipitation, following procedures described previously (46). Whole cell extracts were resuspended in SDS-PAGE sample buffer (62.5 mM Tris-HCl, pH 6.8, 10% glycerol, 2% SDS, 1% 2-mercaptoethanol, 0.0005% bromphenol blue) and boiled for 5 min. Following SDS-polyacrylamide gel electrophoresis and transfer to nitrocellulose, the membrane was probed with antibodies to phospho-p44/p42 at 1:250 (from Cell Signaling, 9101), phospho-p38 at 1:1000 (from Cell Signaling, 4511), phospho-S6 ribosomal protein (Ser235/236) (from Cell Signaling, 2211), GFP at 1:1000 (from Abcam, ab13970), HA at 1:1000 (from Santa Cruz, sc7392), Hog1 (from Santa Cruz, sc6815) and protein A at 1:10,000 (from Sigma-Aldrich, P3775). Immunoreactive species were visualized by enhanced chemiluminescence detection (Pierce) of horseradish peroxidase-conjugated anti-rabbit IgG (Bio-Rad) or anti-chicken IgY (ab97135). All experiments have been repeated at least three times. Immunoblotting signals were quantified with ImageJ software. The dot bar graphs were generated using GraphPad Prism 10, and the bars represent standard deviations. Where indicated, the data were statistically analyzed by *t* test, with *p* < 0.050 considered significant.

## Data availability

All data are contained within the manuscript.

## Conflict of interest

The authors declare that they have no conflicts of interest with the contents of this article.
